# Impacts of Land Use on Soil Nitrogen-Cycling Microbial Communities: Insights from Community Structure, Functional Gene Abundance, and Network Complexity

**DOI:** 10.3390/life15030466

**Published:** 2025-03-14

**Authors:** Junnan Ding, Shaopeng Yu

**Affiliations:** Heilongjiang Province Key Laboratory of Cold Region Wetland Ecology and Environment Research, Harbin University, Harbin 150086, China; wetlands1972@126.com

**Keywords:** land use patterns, Lesser Khingan Mountains, physicochemical properties, nitrogen-cycling functions, soil enzyme activity, co-occurrence pattern

## Abstract

This study investigates the effects of different land-use types (forest, arable land, and wetland) on key soil properties, microbial communities, and nitrogen cycling in the Lesser Khingan Mountains. The results revealed that forest (FL) and wetland (WL) soils had significantly higher soil organic matter (SOM) content compared with arable land (AL), with total phosphorus (TP) being highest in FL and available nitrogen (AN) significantly higher in WL. In terms of enzyme activity, AL and WL showed reduced activities of ammonia monooxygenase (AMO), β-D-glucosidase (β-G), and β-cellobiosidase (CBH), while exhibiting increased N-acetyl-β-D-glucosaminidase (NAG) activity, highlighting the impact of land use on nitrogen dynamics. WL also exhibited significantly higher microbial diversity and evenness compared with FL and AL. The dominant bacterial phyla included Actinobacteriota, Proteobacteria, and Acidobacteriota, with Acidobacteriota being most abundant in FL and Proteobacteria most abundant in WL. Network analysis showed that AL had the most complex and connected microbial network, while FL and WL had simpler but more stable networks, suggesting the influence of land use on microbial community interactions. Regarding nitrogen cycling genes, AOA-*amoA* was most abundant in AL, while AOB-*amoA* was significantly enriched in FL, reflecting the influence of land use on ammonia oxidation. These findings highlight how land-use types significantly affect soil properties, microbial community structures, and nitrogen cycling, offering valuable insights for sustainable land management.

## 1. Introduction

Soil, as a complex biological system, not only provides essential water and nutrients for plant growth but also serves as a critical site for biogeochemical cycling [1]. Microorganisms are the key participants in soil ecological processes, playing significant roles in nutrient cycling, such as decomposing organic matter and litter [2]. The activity and functions of soil microorganisms are significantly influenced by biotic factors (e.g., vegetation characteristics) and abiotic factors (e.g., soil physicochemical properties), which ultimately determine ecosystem stability [3]. Land use changes, driven by a combination of global climate change and human activities, are continuously altering the Earth’s surface landscape patterns [4]. Changes in land use types are often accompanied by variations in ecosystem types, soil microenvironments, vegetation coverage, and nutrient-cycling processes [5]. These changes can impact soil nitrogen input and loss through alterations in vegetation coverage, raising concerns about soil degradation and erosion [6]. Changes in the soil’s structure and microenvironment can influence nitrogen fixation and stability, linking it to global climate change issues. Additionally, variations in soil nutrient-cycling processes can affect plant growth and soil productivity, raising concerns about soil security and ecosystem evolution [7,8,9,10].

Cold-region wetlands are terrestrial ecosystems with unique ecological functions. The characteristics of the soil microbial communities in these wetlands are regulated by the interactions of hydrological conditions, vegetation types, and soil factors [11]. Studies have shown that the rich organic matter and prolonged water saturation in wetland environments provide unique habitats for microorganisms. However, changes in these environmental conditions can significantly alter the structure and functions of soil microbial communities [12]. For example, wetland vegetation degradation or changes can significantly affect biomass, plant cover, and community diversity, indirectly altering soil nutrient supply and physicochemical properties [13]. Understanding how changes in wetland vegetation characteristics and soil factors influence the microbial community’s structure and function is crucial for the management and conservation of cold-region wetland ecosystems. Previous studies have demonstrated that the interactions between wetland vegetation characteristics (e.g., diversity and functions) and soil nutrients undergo significant changes at different stages of degradation [14,15]. However, there is still limited research on the dynamic responses of microbial communities to cold-region wetland environments and the regulatory mechanisms of vegetation and soil factors. Moreover, complex interactions exist among wetland soil microorganisms, forming stable ecological networks that sustain ecosystem functions [16,17]. Positive microbial interactions, such as cooperation, resource sharing, and mutualistic symbiosis, can enhance wetland ecosystem functions [18]. For instance, rhizosphere microorganisms decompose organic matter to provide plants with accessible nutrients while protecting them from pathogens [19]. On the other hand, negative interactions, such as competition or inhibitory effects, can alter microbial community structures and potentially weaken wetland ecosystem stability [20]. Soil microbial diversity is a critical foundation for maintaining the functions of cold-region wetland ecosystems, impacting processes such as biogeochemical cycling, nutrient uptake by plants, and the flow of materials and energy within ecosystems. Through complex interactions and functional collaboration, wetland soil microorganisms form highly intricate networks that are essential for maintaining ecosystem stability [1]. Therefore, exploring the structure, functions, and regulatory mechanisms of soil microbial communities in cold-region wetlands will not only deepen the understanding of wetland ecosystem dynamics but also provide scientific support for wetland conservation and sustainable management.

Soil nitrogen cycling is a vital component of terrestrial ecosystems, encompassing key processes such as ammonification, nitrification, denitrification, and nitrogen fixation [21]. Essentially, nitrogen cycling is a redox reaction network of nitrogen compounds catalyzed by plants, fungi, bacteria, and archaea, with microorganisms serving as the primary drivers of nitrogen transformation and mobility [22,23]. Soil enzyme activities such as those of β-G, CBH, and NAG are crucial for maintaining soil health and driving nutrient cycling, which are fundamental for the functioning of nitrogen-cycling microbial communities [24]. These enzymes facilitate the decomposition of complex organic matter into simpler compounds, thereby releasing essential carbon and nitrogen substrates that support microbial growth and metabolic activities. Specifically, β-G and CBH play key roles in the degradation of cellulose and other polysaccharides, providing carbon sources that enhance soil fertility [25], while NAG is vital for breaking down chitin and other nitrogen-containing polymers, promoting nitrogen mineralization and transformation [26]. The activity of these enzymes is highly responsive to changes in land use; for instance, agricultural practices can disrupt enzyme activity patterns, leading to altered nitrogen-cycling processes and shifts in microbial network complexity [27]. These insights are critical for understanding how different land-use types influence soil ecological functions, as explored in our study on the impacts of land use on soil nitrogen-cycling microbial communities, and they provide valuable guidance for sustainable land management practices. Studies have shown that soil microbial communities are highly sensitive to changes in their habitats, and different land use types can profoundly shape microbial community structure and function by altering soil physicochemical properties and biological environments [28].

Forests, arable land, and wetlands are typical land use types with distinct mechanisms influencing soil nitrogen-cycling microbial communities. Forest soils, with organic matter inputs and minimal human disturbances, typically exhibit high microbial diversity and functional stability, with nitrogen fixation and denitrification as the dominant processes [25]. Arable land soils are influenced by practices such as fertilization and tillage, which significantly alter the soil microenvironment, enhancing ammonification and nitrification processes while potentially increasing nitrogen loss during denitrification [26]. Wetland soils, characterized by prolonged water saturation, provide anaerobic conditions that promote denitrification, reducing nitrate accumulation and loss [27].

Quantitative analysis of microbial functional genes has become a crucial method for understanding the impact of land use changes on soil nitrogen-cycling microbial communities [28]. This approach links functional microbial groups directly to soil properties and nitrogen-cycling processes, offering insights into key microbial processes and mechanisms [29]. However, most existing studies focus on single land use types or specific nitrogen transformation processes. There is relatively little research on the overall impact of forestland, arable land, and wetland land use types on soil nitrogen-cycling microbial communities and their assembly mechanisms [30,31,32]. The objectives of this study are (1) to investigate the response of soil bacterial community composition, diversity, and function to land use changes and identify their driving factors; (2) to examine the differences in the composition and diversity of nitrogen-cycling functional microbial communities under different land use types and to identify the dominant genera involved in nitrogen cycling in each land use type.

## 2. Materials and Methods

### 2.1. Site Description

**Figure 1:**
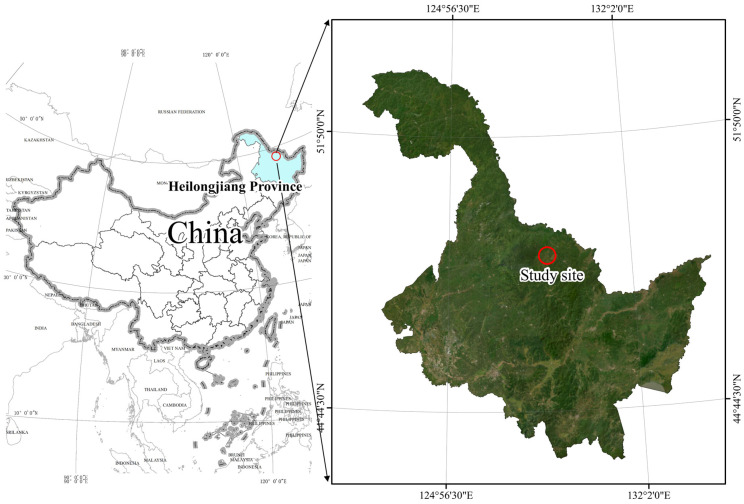
Map showing different land use patterns’ location in the study.

The study area is located in the Hongxing National Nature Reserve (Figure 1), within the Lesser Khingan Mountains, situated in the northeast of Heilongjiang Province, China (128°21′40″~128°53′30″ E and 48°41′20″~49°11′00″ N). Covering an area of 111,995.3 hectares, the reserve experiences a northern temperate continental monsoon climate, characterized by an annual average temperature of −0.7 °C, an average precipitation range of 500–610 mm, and an average relative humidity of 71.1%. The Hongxing National Nature Reserve is notable for its high biodiversity, encompassing a range of ecosystems including typical water, swamp, meadow wetland, shrub, and forest vegetation. The reserve is home to 1929 species, among which 885 are plant species and 197 are bryophytes distributed across 49 families. Additionally, the reserve contains 38 species of ferns from 11 families and 650 species of seed plants from 88 families [33]. The wetland park within the reserve is categorized into seven types of wetlands: rivers, flood plains, herbs, mosses, swamp meadows, shrub swamps, and forest swamps. The primary landscape of the park is dominated by herb and swamp meadow wetlands, which are surrounded by agricultural lands.

### 2.2. Experimental Design

The vegetation characteristics of the soil sampling sites are classified into three distinct land use types. FL is previously degraded wetlands, now characterized by primary forests dominated by mixed coniferous and broad-leaved trees; its soil type is mainly Alfisols. AL is formerly wetlands that were converted into agricultural land 30 years ago. The primary crop is soybean (*Glycine max* L.), cultivated continuously on an annual cycle. Agricultural practices involve tillage, fertilization, and field management in line with local traditions; its soil type is mainly Mollisols. WL is once arable land that is now dominated by plant species such as *Deyeuxia angustifolia*, *Carex lehmanii*, *Carex meyeriana*, *Carex appendiculata*, *Caltha palustris*, *Cyamus flavicomus*, and *Pedialaris langiflora*; its soil type is mainly Vertisols. Agricultural machinery is used for tillage in late April, followed by soybean sowing. Fertilization and field management follow local practices, with the crop harvested in late September. The land remains idle from late October to mid-April of the following year. These land use types reflect distinct ecological and management histories, making them suitable for examining the impacts of land use changes on soil properties and microbial communities [34,35].

### 2.3. Sample Collection

The experiment was conducted from 20 August to 3 September 2024 at the Key Laboratory of Wetland Ecology and Environmental Research of Harbin University (Harbin, China) and the Hongxing National Nature Reserve in Heilongjiang Province, China. Based on the land use characteristics of the nature reserve, three types of 100 × 100 m quadrats were established, representing meadow wetlands, forests, and surrounding soybean arable land. In each quadrat, five sampling points were selected using an “S” pattern. Soil samples were collected from a depth of 0–0.20 m at each point using a 5 cm soil shovel. The collected samples were mixed to form a composite sample, and each composite sample was replicated three times. Fresh soil samples from each quadrat were passed through a 2 mm sieve to remove root residues. During sampling, a portion of the fresh soil was stored at −20 °C in a refrigerator for the determination of soil NH_4_^+^-N and NO_3_^−^-N enzyme activity as well as the abundance of soil microorganisms. The remaining soil samples were air-dried, ground separately, and sieved through 0.85 mm and 0.15 mm aperture sieves. These processed samples were then stored in self-sealing bags for the analysis of soil pH and organic matter physicochemical properties.

### 2.4. Soil Chemical Analysis

Soil chemical properties were analyzed using established laboratory methods. Soil pH was measured using a pH meter to determine the acidity or alkalinity. The SOM content was determined by the Walkley–Black method, where soil samples were oxidized using potassium dichromate and concentrated sulfuric acid under acidic conditions. After oxidation, the remaining dichromate was titrated with a reducing agent (e.g., sodium oxalate), and the SOM content was calculated based on dichromate consumption and a standard curve, expressed as a percentage of soil weight [36]. The soil bulk density (SBD) was measured using the core method: soil samples of a fixed volume were collected with a core sampler, oven-dried at 105 °C to a constant weight, and then the dry mass was divided by the core volume to calculate bulk density. Total nitrogen (TN) was analyzed using concentrated sulfuric acid digestion followed by the Kjeldahl method [37]. The content of AN in the soil samples was determined using the alkali hydrolyzable diffusion method [38]. Phosphorus content was determined using two approaches: The content of TP was analyzed by digesting soil with HClO_4_ and H_2_SO_4_ followed by molybdenum antimony anti-colorimetry. Available phosphorus (AP) was extracted using NaHCO_3_ and measured with the same colorimetric method [39]. Potassium content was assessed by determining total potassium (TK) through soil digestion with nitric, perchloric, and hydrofluoric acids, followed by flame photometry or atomic absorption spectrophotometry [40]. Available potassium (AK) was extracted using 1 M ammonium acetate (pH 7.0) and quantified with similar spectrometric techniques. These comprehensive analyses provided essential insights into soil chemical properties, aiding in understanding their influence on microbial community structures and functions [41]. The soil NH_4_^+^-N and NO_3_^−^-N were determined using indophenol blue colorimetry and phenol disulfonic acid colorimetry, respectively [42].

### 2.5. Determination of Soil Enzyme Activity

Fluorescent microplate enzyme detection technology was employed to measure the activities of soil β-G, CBH, and NAG, with 4-hydroxymethyl-7-coumarin (MUB) used as the standard [43]. Each treatment included sample wells, blank controls, negative controls, quenching controls, and reference controls arranged on a 96-well plate. Following a 4 h incubation at 25 °C in the dark, fluorescence was measured using a microplate reader at excitation and emission wavelengths of 365 nm and 450 nm, respectively. Soil activities of ammonia monooxygenase (AMO), hydroxylamine oxidoreductase (HAO), and nitrite oxidoreductase (NXR) were analyzed using an ELISA kit provided by Jiangsu Meibiao Biotechnology Co., Ltd. (Yancheng, Jiangsu, China).

The enzyme activity was defined as the amount of ammonium ion (mg) released from the hydrolysis of one gram of soil at 37 °C over 24 h. Soil denitrification enzyme activities, including nitrate reductase (NR) and nitrite reductase (NiR), were measured using the benzenesulfonic acid–acetic acid–α-naphthylamine colorimetric method [44]. NR activity was expressed as the milligrams of NO_2_^−^ generated from 1 kg of soil at 30 °C in 24 h, while NiR activity was represented by the milligrams of NO_2_^−^ reduced under the same conditions [45]. The activities of L-leucine aminopeptidase (LAP) and NAG were determined following a modified protocol from Bob Sinsabaugh’s lab [46]. Crude enzyme solutions were prepared with a pH 5 buffer. LAP activity was measured using 5 mM leucine p-nitroaniline as a substrate, while NAG activity employed 2 mM pNP-β-acetylglucosaminide. Controls were included, and enzymatic activities were quantified colorimetrically using a microplate reader. The activity unit was defined as the amount of substrate (mg) hydrolyzed per gram of dry matter per hour. Soil NH₄⁺-N and NO₃⁻-N concentrations were determined using indophenol blue and phenol disulfonic acid colorimetric methods, respectively [42]. Soluble organic nitrogen was extracted with 0.5 mol L⁻¹ K₂SO₄ and analyzed with a Total Organic Carbon Analyzer (TOC-VcPH + TNM-1, Shimadzu Inc., Kyoto, Japan) following the method described by Edwards et al. [47]. Microbial biomass nitrogen (MBN) was measured using an improved chloroform fumigation–extraction method. Organic nitrogen in fumigated and non-fumigated extracts was quantified with a Total Organic Carbon Analyzer, and MBN was calculated by dividing the difference in organic nitrogen between the fumigated and non-fumigated samples by 0.54 [48].

### 2.6. Soil DNA Extraction and High-Throughput Assay

Genomic DNA from soil microorganisms was extracted using the Omega E.Z.N.A. DNA Kit (Omega Bio-Tek, Norcross, GA, USA). The quality of the extracted DNA was assessed through agarose gel electrophoresis. Polymerase chain reaction (PCR) was conducted on a GeneAmp 9700 PCR system (Applied Biosystems, Thermo Fisher Scientific, Waltham, MA, USA) to amplify the V3-V4 region of the bacterial 16S rRNA gene using universal primers 515F (5’-GTGCCAGCMGCCGCGGTAA-3′) and 907R (5’-CCGTCAATTCMTTTRAGTTT-3′). The PCR products were quantified with a QuantiFluor^®^-ST fluorometer (Promega, Madison, WI, USA) and adjusted for sequencing. High-throughput sequencing was performed on the Illumina HiSeq 2500 PE250 platform (San Diego, CA, USA) by Shanghai Meiji Biotechnology Co., Ltd. (Shanghai, China) [49].

The raw paired-end reads generated from the Illumina platform were demultiplexed and quality-filtered using a combination of Cutadapt and DADA2 (within the QIIME2 environment) to remove adapter contamination, low-quality reads, and chimeric sequences. After denoising and merging paired-end reads, Amplicon Sequence Variants (ASVs) were identified at 100% sequence similarity. Taxonomic assignment of ASVs was carried out by aligning representative sequences against the SILVA rRNA gene database using a Naïve Bayes classifier. Singletons and rare ASVs were removed to minimize potential sequencing artifacts. Alpha diversity indices (e.g., Shannon, Chao1) and beta diversity metrics (Bray–Curtis dissimilarity) were calculated using QIIME2 and R software (R Core Team, Vienna, Austria). Principal Coordinates Analysis (PCoA) was used to visualize differences in microbial communities, and PERMANOVA was conducted to assess the significance of community composition differences among samples.

Real-time quantitative PCR (RT-qPCR) was performed using 0.25 g of fresh soil samples. Genomic DNA was extracted with the Mo Bio PowerSoil^®^ DNA Extraction Kit (Qiagen, Germany), and the quality and concentration of the extracted DNA were assessed using a NanoDrop Spectrophotometer (NanoDrop Technologies, Wilmington, DE, USA), following the method described by Edwards et al. [47]. RT-qPCR was carried out on an ABI7500 Real-Time PCR System (Applied Biosystems, Foster City, CA, USA) using the SYBR® Premix Ex Taq Kit (Takara, Shiga, Japan) to quantify microbial genes associated with nitrogen-cycling processes.

Targeted genes included those involved in ammonification (*gdh*), nitrification (AOA-*amoA* and AOB-*amoA*), denitrification (*nirS*, *nirK*, and *nosZ*), nitrogen fixation (*nifH*), and nitrate dissimilatory reduction (*napA*). Each qPCR reaction was performed in a total volume of 25 µL, consisting of 1 µL of DNA template, 12.5 µL of SYBR^®^ Premix Ex TaqTM, 0.5 µL each of forward and reverse primers, 0.5 µL of ROX Reference Dye II (50×), and ddH₂O to complete the volume. Primer sequences used for amplifying the nitrogen cycle functional genes are listed in Table S1.

### 2.7. Statistical Analysis

This study utilized R software version 4.0.2 for data processing and analysis, employing the following specific methods and R packages. Differential analysis of soil factors across different land use types was performed using the ggplot2 and ggpubr packages, with significance testing to assess the differences between groups. For correlation analysis, both Pearson correlation and Spearman rank correlation were used to examine the relationships between microbial taxa and soil environmental factors, identifying microbial groups strongly correlated with key soil properties. The ggplot2 and ggpubr packages were used to perform differential analysis of soil factors across different land use types. These packages enabled data visualization and significance testing, allowing for clear graphical representations of the relationships between soil factors and land use [50,51]. The VennDiagram package was utilized to analyze the shared and unique operational taxonomic units (OTUs) across different degrees of degradation, providing insights into the differences in microbial community composition at varying stages of land degradation. The OTU analysis was conducted using the Meiji Biotechnology Cloud Platform (https://www.majorbio.com/web/www/index, accessed on 16 November 2024) [52]. Prior to diversity analysis, the OTU table was rarefied to 90% of the minimum sample sequencing depth to normalize sequencing effort across samples and mitigate potential biases caused by variations in sequencing depth. To analyze the interactions among microbial communities, undirected correlation networks were constructed using the igraph package. The Gephi software (version 0.9.2) was used to visualize the network diagrams, providing an intuitive way to interpret complex microbial interactions [53]. Network topology features, including characteristic path length, number of connections, number of nodes, clustering coefficient, network density, and average connectivity, were calculated using the Network Analyzer tool in Gephi to examine their differences across various stages of degradation [54,55]. Robustness was calculated by simulating the removal of 10% of nodes randomly and assessing the change in natural connectivity before and after removal. Vulnerability was quantified by sequentially removing each node and measuring the decline in network efficiency. Community stability was evaluated based on the modularity index, calculated using Newman’s algorithm, and the redundancy of keystone taxa, determined by the impact of node removal on network connectivity. These network parameters offered valuable insights into the structural complexity and stability of microbial communities across the study area. Additionally, correlation-based methods were applied to further investigate the relationships between microbial taxa and soil environmental factors. Pearson’s and Spearman’s correlations were used to identify significant microbial taxa that might be strongly correlated with key soil properties, shedding light on their ecological roles in soil ecosystems [56].

## 3. Results

### 3.1. Effects of Soil Physicochemical Properties Under Land Use Patterns

**Figure 2:**
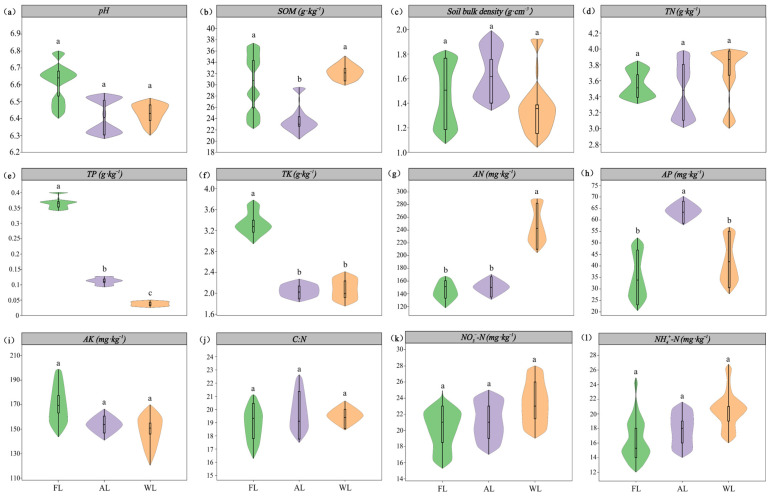
Soil properties under different land use patterns. Abbreviations: (**a**) pH, soil pH values. (**b**) SOM, soil organic matter. (**c**) SBD, Soil bulk density. (**d**) TN, total nitrogen. (**e**) TP, total phosphorus. (**f**) TK, total potassium. (**g**) AN, available nitrogen. (**h**) AP, available phosphorus. (**i**) AK, available potassium. (**j**) C:N, the ratio of total C and total N. (**k**) NO_3_^−^-N, nitrate nitrogen. (**l**) NH_4_^+^-N, ammonium nitrogen. FL, forestland. AL, arable land. WL, wetland. Significant differences among treatments are indicated in different lowercase letters (*p* < 0.05).

The changes in the physical and chemical properties of soil samples under different land use types are shown in Figure 2. The content of SOM in FL and WL soils was significantly higher than that in AL soils, increasing by 27.76% and 24.02%, respectively (*p* < 0.05). The content of TP in FL soil samples was significantly higher than that in AL and WL soils, increasing by 64.88% and 98.81%, respectively (*p* < 0.05). The content of AN in WL soils was significantly higher than that in FL and AL soils (*p* < 0.05). The content of AP in AL soil samples was significantly higher than that in FL and WL soils, increasing by 46.17% and 37.76%, respectively (*p* < 0.05). However, there were no significant differences in soil pH, SBD, TN, AK, C:N ratio, NH₄⁺-N, and NO₃^−^-N.

### 3.2. Composition of Soil Microbial Community

**Figure 3:**
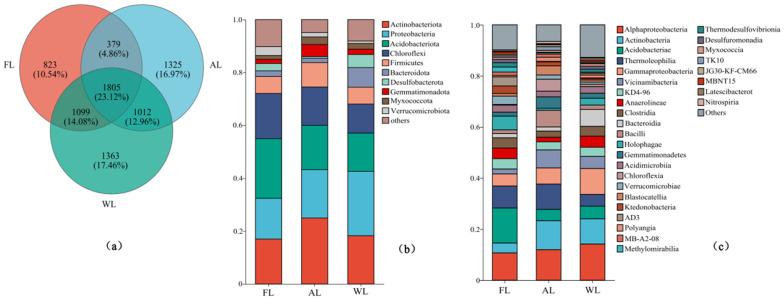
Composition of soil microbial communities under different land use patterns. (**a**) Venn diagram of soil microorganisms based on the level of OTUs. (**b**) Relative abundance of soil bacterial communities under different land uses at the phylum level. (**c**) Relative abundance of soil bacterial communities under different land uses at the class level. Abbreviations: FL, forestland. AL, arable land. WL, wetland.

The intersection of OTU sequences exhibiting over 96% similarity with the soil bacterial communities associated with each treatment is depicted as a Venn diagram (Figure 3). The abundance of bacterial OTUs significantly differed among land use types (*p* < 0.05), with wetland (WL) showing the highest abundance. A total of 1363 OTUs were exclusively found in wetland soil bacteria, representing a significant proportion of the overall OTU sequences (*p* < 0.05, Figure 3a). Figure 3b illustrates that while the composition of the top ten dominant bacterial species was comparable across the different soil samples, the abundance levels of these bacterial communities varied significantly among the different land use patterns. At the taxonomic phylum level for the examined soil samples, the dominant bacterial groups were Actinobacteriota, Proteobacteria, Acidobacteriota, Chloroflexi, and Firmicutes. Acidobacteriota, one of the predominant microbial phyla in forestland soils, exhibited a relative abundance of 22.57% within the total bacterial community (Figure 3b). Similarly, the relative abundance of Actinobacteriota, Thermoleophilia, and Vicinamibacteria was significantly higher in arable land compared with other land use types (*p* < 0.05, Figure 3b,c). In contrast, the wetland exhibited the highest relative abundance of Proteobacteria, Alphaproteobacteria, and Gammaproteobacteria (*p* < 0.05, Figure 3b,c).

### 3.3. Analysis of Changes in Soil Microbial Alpha Diversity

**Figure 4:**
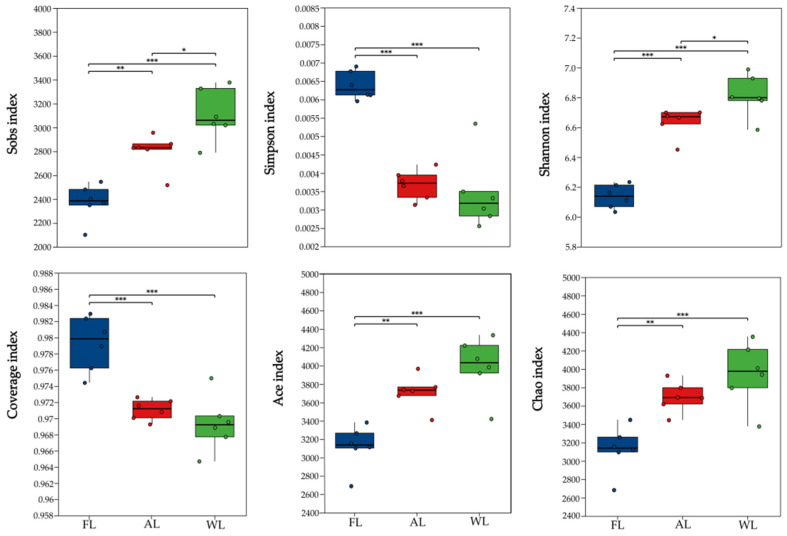
Diversity index of soil bacterial communities in different land use patterns. * *p* < 0.05; ** *p* < 0.01; and *** *p* < 0.001. Abbreviations: FL, forestland. AL, arable land. WL, wetland.

The diversity index analysis shown in Figure 4 indicates that the Sobs, Shannon, Ace, and Chao indices of soil microbial communities in the WL sites were significantly higher than those in the AL and FL sites (*p* < 0.05, <0.001). The unique hydrological conditions of wetland environments (e.g., periodic flooding) and abundant organic matter provide a stable habitat for diverse microbial communities. Additionally, the growth of specialized microbial taxa under anaerobic conditions further enhances community richness. The high Shannon index in wetlands suggests not only a rich microbial community but also a relatively even distribution of species. The results of the Simpson and Coverage indices for soil microbial communities indicate that FL sites had significantly higher Simpson and Coverage indices compared with AL and WL sites (*p* < 0.001). The higher Simpson and Coverage indices in FL suggest a more even distribution of microbial communities in forest soils. In contrast, the lowest Simpson index in WL implies the presence of dominant species within the wetland soil microbial community.

### 3.4. Correlation Analysis of Environmental Factors

**Figure 5:**
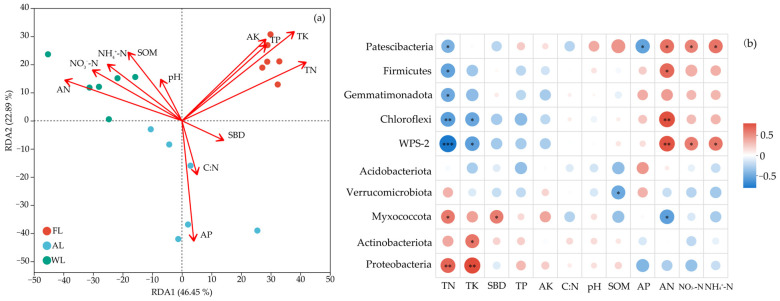
Environmental factor correlation analysis. The redundancy analysis (RDA, (**a**)) and heat map (**b**) of the soil bacterial community at the OTU level. * *p* < 0.05, ** *p* < 0.01, and *** *p* < 0.001. Abbreviations: pH, soil pH values. SOM, soil organic matter. SBD, Soil bulk density. TN, total nitrogen. TP, total phosphorus. TK, total potassium. AN, available nitrogen. AP, available phosphorus. AK, available potassium. C:N, the ratio of total C and total N. NO_3_^−^-N, nitrate nitrogen. NH_4_^+^-N, ammonium nitrogen. FL, forestland. AL, arable land. WL, wetland.

Figure 5a illustrates the effects of environmental factors on soil characteristics in FL, WL, and AL sites. RDA1 and RDA2 collectively explain 69.34% of the variation in the data, indicating that these environmental factors strongly influence community distribution. FL showed a positive correlation with TN, TP, TK, and AK while exhibiting a negative correlation with C:N and AP. AL was positively correlated with AP and C:N but negatively correlated with TN and SOM. WL was positively correlated with SOM, NH₄⁺-N, and NO₃⁻-N, while negatively correlated with pH and SBD. The correlation analysis between different environmental factors and microbial phyla is shown in Figure 5b. Proteobacteria exhibited a significant positive correlation with AN (*p* < 0.001) and NH₄⁺-N (*p* < 0.05) but showed a negative correlation with TN and TK. Firmicutes, Actinobacteriota, and Gemmatimonadota were positively correlated with AP. Actinobacteriota were negatively correlated with SOM, pH and TP (*p* < 0.05). Gemmatimonadota were negatively correlated with TK (*p* < 0.01) and TP (*p* < 0.001). Nirospirota were positively correlated with TK, AK, and TP but negatively correlated with AP (*p* < 0.001). Acidobacteriota showed a positive correlation with TN (*p* < 0.001) and TK (*p* < 0.01) and a negative correlation with AN (*p* < 0.05). Chloroflexi displayed a significant positive correlation with TN (*p* < 0.05) and a significant negative correlation with AN (*p* < 0.001). Verrucomicrobiota showed a positive correlation with TN (*p* < 0.001) and AK (*p* < 0.05) and a significant negative correlation with AN (*p* < 0.05). Desulfobacterota were positively correlated with AN (*p* < 0.01) and SOM (*p* < 0.05), and significantly negatively correlated with AP (*p* < 0.05).

### 3.5. Effects of Land Use Types on Soil Nitrogen Mineralization, Nitrification, and C-N Cycle-Related Enzymes Activity

**Figure 6:**
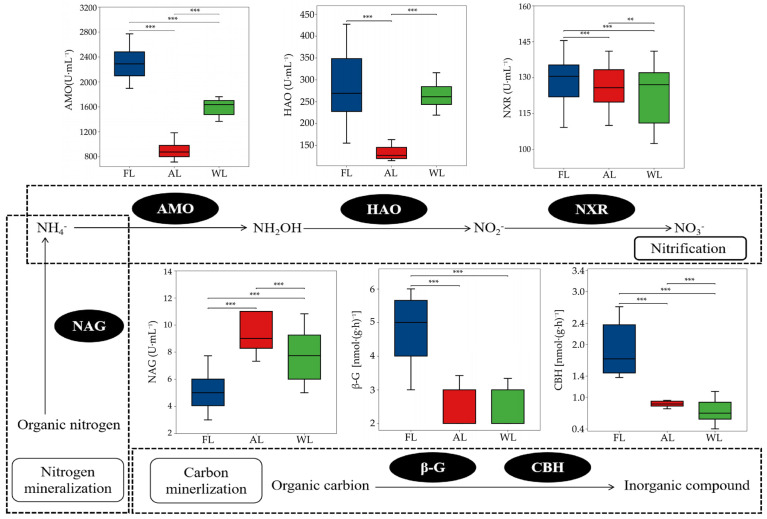
Activity of C and N cycle-related enzymes of soil under different land use patterns. ** *p* < 0.01, *** *p* < 0.001. Abbreviations: FL, forestland. AL, arable land. WL, wetland. AMO, ammonia monooxygenase activity. HAO, hydroxylamine oxidoreductase activity. NXR, nitrite oxidoreductase activity. NAG, N-acetyl-β-D-glucosaminidase activity. β-G, β-D-glucosidase activity. CBH, β-cellobiosidase activity.

The soil nitrogen mineralization process across different land types is shown in Figure 6. Compared with FL, both AL and WL significantly reduced AMO activity by 61.28% and 38.71%, respectively (*p* < 0.001). In the soil nitrogen nitrification process, AL significantly reduced the activities of β-G and CBH compared with FL (*p* < 0.001). The activities of HAO were increased in AL compared with FL and WL. However, AL significantly reduced HAO activity by 52.43% and 52.69%, respectively, compared with FL and WL (*p* < 0.001). The activities of NXR did not show significant differences across the different land types. Compared with FL, AL and WL resulted in significant increases in NAG activity, by 40.72% and 28.37%, respectively (*p* < 0.001).

### 3.6. Effects of Major Control Factors on Soil Microbial Properties

**Figure 7:**
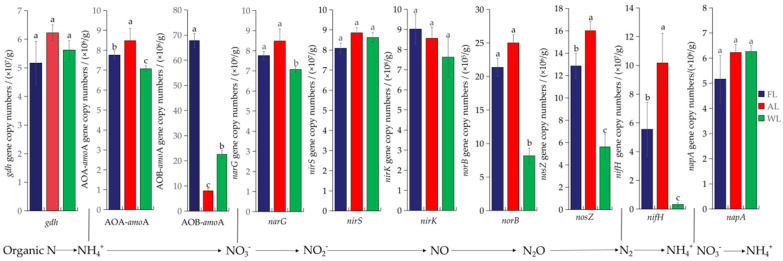
Abundance of nitrogen-cycling functional genes. Different letters above the bars indicate statistical differences among treatments at the significance level of *p* < 0.05. Abbreviations: FL, forestland. AL, arable land. WL, wetland.

The Figure 7 demonstrates that different land-use types (FL, AL, and WL) significantly influence the abundance of nitrogen-cycling functional genes, displaying distinct variations and specific trends among the treatments as well as notable percentage differences in abundance. The abundance of the ammonification functional gene *gdh* shows little variation, with no significant differences observed across the three treatments. The ammonia-oxidizing functional gene AOA-*amoA* exhibits the highest abundance in AL, which is 8.49% and 16.62% higher than in FL and WL, respectively, indicating that arable land strongly supports ammonia-oxidizing archaea. In contrast, AOB-*amoA* is significantly enriched in FL, with an increase of 88.08% compared with AL and 66.77% compared with WL, indicating that forest ecosystems strongly promote the proliferation of ammonia-oxidizing bacteria. Compared with the WL, the abundance of the functional gene *narG* under the FL and AL was significantly increased by 0.91% and 16.62%, respectively (*p* < 0.05). Among denitrification functional genes, the abundance of *nirS*, *nirK*, and *napA* was consistent across FL, AL, and WL, showing no significant differences. The abundance of the nitric oxide reduction functional gene *norB* was significantly higher in AL, with an increase of 14.56% compared with FL and an increase of approximately 67.36% compared with WL, reflecting enhanced nitric oxide reduction activity in agricultural soil. Similarly, the nitrous oxide reduction functional gene *nosZ* shows the highest abundance in AL, with a 19.68% increase compared with FL and a 64.93% increase compared with WL, demonstrating that wetland soil strongly supports nitrous oxide-reducing microorganisms. The nitrogen fixation functional gene *nifH* was more abundant in FL and AL compared with WL, with an increase of 94.41% and 96.95%, respectively, and showed significant differences (*p* < 0.05).

### 3.7. Effects of Land Use Types on Microbial Co-Occurrence Patterns

**Figure 8:**
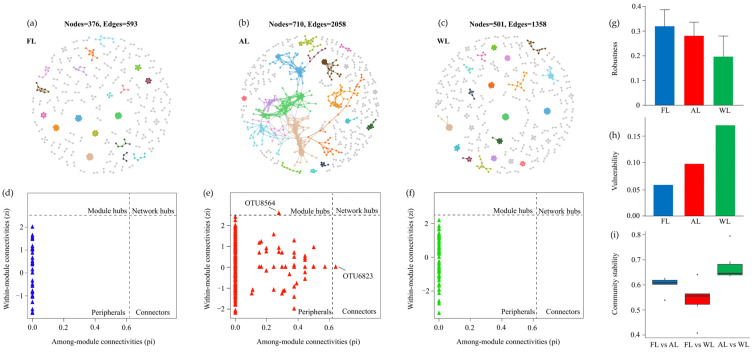
Co-occurrence network of soil bacteria dominant OTUs. Note: Network nodes and links are colored according to module properties, with large modules (≥5 nodes) shown in different colors and smaller modules shown in gray. Abbreviations: FL, forestland. AL, arable land. WL, wetland. (**a**–**c**), co-occurrence network. (**d**–**f**), the network topology parameters. (**g**–**i**), the soil bacteria dominant OTUs analysis for robustness, vulnerability, and community stability analysis.

The network analysis revealed significant differences in microbial community networks among FL, AL, and WL in terms of node count, edge count, and topological structure (Figure 8). The FL network consisted of 376 nodes and 593 edges, indicating a sparse network with relatively low connectivity and complexity in microbial interactions (Figure 8a). The AL network showed the highest node and edge counts, reaching 710 and 2058, respectively, reflecting more extensive microbial interactions and significantly increased network density in agricultural environments (Figure 8b). The WL network, with 501 nodes and 1358 edges, displayed moderate complexity (Figure 8c), lying between FL and AL, suggesting balanced microbial community structures in wetland soils. Further analysis of network topology parameters highlighted notable differences among the three land-use types. FL exhibited a high proportion of peripheral nodes with fewer module hubs and network hubs, indicating that most microbial interactions were isolated or had low connectivity (Figure 8d). In contrast, AL had the highest number of network hubs and module connectivity, suggesting the presence of more critical microbial taxa in maintaining the network structure, with enhanced overall connectivity (Figure 8e). WL displayed a higher proportion of connector nodes, indicating stronger cross-module interactions among microbial taxa, which contribute to maintaining network functionality in wetland environments (Figure 8f). Regarding robustness and vulnerability, the FL network exhibited the highest robustness, demonstrating enhanced tolerance to the loss of individual nodes due to its high connectivity (Figure 8g,h). However, WL showed higher vulnerability, indicating that its highly connected network is more susceptible to collapse under large-scale disturbances (Figure 8h). The FL and WL networks exhibited lower robustness but also lower vulnerability, reflecting greater resilience, as their network structures were less affected by the loss of individual nodes (Figure 8g,h). Community stability analysis revealed that FL and WL networks had higher stability, reflecting smaller dynamic changes in microbial communities in natural ecosystems (Figure 8i). In contrast, the AL network showed lower stability, likely due to frequent disturbances caused by agricultural activities. The FL network displayed low complexity, low connectivity, and high stability, representing localized microbial interactions in forest soils. The AL network had high complexity and connectivity, coupled with increased robustness and vulnerability, indicating dynamic and extensive interactions. The WL network exhibited moderate complexity and connectivity, reflecting the adaptability and balance of microbial communities in wetland environments. These results provide clear evidence of the impacts of different land-use types on microbial community network structures.

## 4. Discussion

### 4.1. Impacts of Land-Use Patterns on Soil Bacterial Community Structure

This study systematically reveals the significant impacts of different land-use types on soil physicochemical properties and microbial community structures, underscoring the complex interrelationships between them. The markedly elevated soil organic matter content in FL and WL not only highlights the critical role of natural vegetation in soil carbon storage but also illustrates, from a microbial ecological perspective, the positive driving effect of high-organic-matter environments on microbial communities [57]. In wetland soils, the number of operational taxonomic units (OTUs) reaches 5279, with approximately 17.46% of these OTUs being unique to wetlands, demonstrating the irreplaceable ecological value of wetlands for providing distinctive microbial habitats [58]. This finding is closely linked to the substantial accumulation of organic matter in wetlands, relatively stable water conditions, and anaerobic processes, thereby further corroborating the essential role of wetlands in sustaining high microbial diversity and associated ecological functions [59]. By contrast, the relatively low SOM content and higher level of AP in AL reflect the profound reshaping of soil physicochemical characteristics by intensive agricultural activities. While fertilization and tillage can enhance nutrient availability in the short term, these practices may also diminish soil structure and microbial diversity or favor particular functional microbial communities [60]. For example, the relatively high abundance of Actinobacteriota, Thermoleophilia, and Vicinamibacteria in agricultural soils indicates that these groups are well adapted to the rapid utilization of exogenous soil nutrients and the decomposition of residual organic matter [61]. However, this convergence or functional specialization of microbial communities may partially constrain the overall stability and functional redundancy of soil ecosystems [62].

The high level of AN in wetland soils is closely associated with the distinctive anaerobic conditions found in wetlands. Anaerobic environments inhibit nitrogen volatilization and accelerate microbial processes such as denitrification, resulting in a marked dominance of Proteobacteria, particularly Alphaproteobacteria and Gammaproteobacteria, in wetland soils [63]. These groups play vital roles in the decomposition of organic matter and nutrient cycling, reinforcing the central function of wetlands in regulating carbon, nitrogen, and other essential nutrient cycles [64]. In comparison, the highest relative abundance of Acidobacteriota (22.57%) in FL aligns with the typically acidic, low-nutrient conditions in forest ecosystems. These microbes not only survive effectively in acidic environments but also play an important part in organic matter cycling and the maintenance of nutrient stability in forest soils [65]. Notably, although land-use types significantly affect key soil nutrient indicators such as SOM, TP, and AP, there are no significant differences in soil pH, SBD, and TN across the different land-use types. This suggests that these physical indicators are more likely governed by long-term environmental factors such as geological background and climate conditions rather than short-term land management practices [66]. Meanwhile, the high sensitivity of microbial communities to variations in soil nutrient levels and organic matter further underscores the role of soil microorganisms as “sensors” of ecosystem functioning [67].

### 4.2. Effect of Land Use on Soil Bacterial Community Diversity

Analysis of alpha diversity indices reveals that wetland sites support significantly higher microbial richness (Sobs, Ace, and Chao) and diversity (Shannon) compared with AL and FL. This finding is consistent with the specialized conditions in wetlands—periodic flooding, high moisture retention, and abundant organic matter—that collectively create a stable yet heterogeneous habitat for various microbial taxa. The availability of anaerobic niches further encourages the growth of specialized microorganisms, including certain classes of Proteobacteria (e.g., Alphaproteobacteria and Gammaproteobacteria), methanogenic archaea, and other anaerobic or facultative anaerobic groups. These factors together contribute to the overall high alpha diversity in wetlands, as evidenced by the elevated Shannon index, which also suggests a relatively balanced distribution of species within the wetland microbial community. However, the lower Simpson index in WL indicates the presence of certain dominant taxa that may be disproportionately abundant under wetland conditions. In contrast, FL soils exhibit higher Simpson and Coverage indices, reflecting a more even community structure and suggesting that the sampling thoroughly captured the majority of the microbial taxa. The relatively stable environment and consistent litter input in forest ecosystems could explain this evenness, as it fosters a variety of microbial groups—especially Acidobacteriota—in a more homogeneous nutrient context. On the other hand, AL soils, which experience frequent disturbances from tillage and chemical inputs, may show reduced richness and an intermediate community evenness, as certain fast-growing or disturbance-tolerant taxa (e.g., Actinobacteriota, Thermoleophilia) become more dominant.

The alpha diversity metrics underscore how distinct land use patterns, with their respective microenvironmental conditions and disturbance regimes, shape the soil microbial community composition and structure. Wetlands provide a high-diversity habitat with several dominant taxa adapted to waterlogged, anaerobic conditions, whereas forests maintain a more even distribution of microbial species. Agricultural soils, subject to periodic tillage and fertilization, fall in between these extremes in terms of alpha diversity and community evenness. This insight emphasizes the importance of preserving or restoring natural habitats such as wetlands and forests, as well as adopting sustainable agricultural practices, to maintain or enhance soil microbial diversity and associated ecosystem functions.

The RDA revealed selected environmental factors of the variation in soil characteristics across FL, WL, and AL, highlighting the key role of soil physicochemical properties in shaping microbial communities under different land-use types [68]. The FL is positively correlated with TN, TP, TK, and AK, reflecting higher nutrient contents due to litter inputs and minimal disturbances [69]. However, FL shows negative correlations with C:N and AP, indicating lower phosphorus availability and narrower C:N ratios despite high nutrient reserves [70]. In AL, intensive practices such as fertilization and tillage increase AP and C:N but deplete SOM and TN, often due to accelerated mineralization and nitrogen loss over time [71]. The WL is positively correlated with SOM, NH₄⁺-N, and NO₃⁻-N, while the negative correlations with soil pH and SBD reflect wetland hydrological processes that enhance organic matter retention and higher inorganic nitrogen concentrations [72,73].

The correlation analysis between microbial phyla and soil properties sheds light on how individual taxa adopt diverse ecological strategies in response to soil nutrient availability and form (Figure 5b). The ecological strategy of Proteobacteria may have evolved, suggesting these microorganisms now benefit from soils rich in specific inorganic nitrogen sources such as ammonium nitrogen rather than relying solely on the total nutrient content of the soil [74]. This shift highlights their ability to adapt to inorganic nitrogen, especially ammonium, indicating that they may prioritize easily accessible nitrogen sources over total nitrogen content [75]. This aligns with Proteobacteria’s diverse metabolic functions, particularly in the nitrogen cycle, where they efficiently utilize inorganic nitrogen sources through processes like nitrate reduction or ammonification [76]. Studies show that Proteobacteria exhibit significant metabolic flexibility in nitrogen-rich environments, enabling them to dominate by converting soil nitrogen into bioavailable forms [77]. Their ability to quickly adapt to changes in nitrogen availability and enhance absorption efficiency supports their competitiveness in rapidly changing environments [78]. Agricultural and land management practices such as fertilization further alter soil nitrogen availability, providing more favorable conditions for Proteobacteria, especially in terms of ammonium adaptation [79]. In contrast, other microbial groups may rely on stable nitrogen sources or more complex nitrogen-cycling processes, leading to different ecological strategies [80]. The reliance of Proteobacteria on inorganic nitrogen, particularly ammonium, is closely linked to their metabolic flexibility, allowing them to gain a competitive advantage in soils with fluctuating nitrogen availability without needing high concentrations of total nitrogen or potassium [81,82].

Firmicutes, Actinobacteriota, and Gemmatimonadota showed positive correlations with AP, indicating that these groups may have a growth advantage in phosphorus-rich soil environments. Actinobacteriota, known for their strong organic matter decomposition abilities and diverse metabolic pathways, may release organic phosphorus through organic matter decomposition and subsequently utilize phosphorus in the soil [83]. Gemmatimonadota may adapt to phosphorus-rich soils through unique metabolic pathways, allowing them to thrive and even compete effectively in nutrient-rich environments [84]. However, Actinobacteriota’s negative correlations with SOM, pH, and TP suggest they may prefer soils with lower organic matter and slightly acidic to neutral pH values. This could indicate their ability to thrive in environments with slower organic matter decomposition or to outcompete other microorganisms for organic carbon and phosphorus [85]. Moreover, their negative correlations with pH and total phosphorus suggest they may be more adaptable to soils with lower phosphorus concentrations, where they can effectively utilize other available nutrients [86]. The negative correlations between Gemmatimonadota, TK, and TP indicate their adaptation to low-nutrient environments. Gemmatimonadota are often found in nutrient-poor soils, particularly those with low potassium and phosphorus levels, suggesting their ability to utilize lower concentrations of these mineral elements and occupy niches in resource-limited soils [87,88]. In contrast, Nitrospirota were positively correlated with TK, AK, and TP, suggesting that they may be more reliant on mineral elements like potassium and phosphorus, especially in the nitrogen cycle, where they utilize these nutrients to enhance metabolic activity. However, their negative correlation with AP indicates that Nitrospirota may not depend on readily available phosphorus but instead adapt to less bioavailable phosphorus forms in the environment [89]. This adaptation may be central to their role in the nitrogen cycle. Acidobacteriota were positively correlated with TN and TK, suggesting they thrive in environments with higher nitrogen and potassium concentrations, potentially utilizing these abundant nutrients for metabolic activities [90]. However, their negative correlation with AN suggests that Acidobacteriota are better suited to environments with less accessible nitrogen, likely adapting to low-nutrient, low-pH soils where they utilize more complex nitrogen forms for growth and metabolism [91,92]. Chloroflexi were positively correlated with TN, suggesting they thrive in nitrogen-rich environments, potentially utilizing more stable nitrogen sources rather than readily available nitrogen forms, as indicated by their negative correlation with AN. Similarly, Verrucomicrobiota showed a strong positive correlation with TN and AK, indicating their adaptation to nitrogen- and potassium-rich environments [93]. Their negative correlation with AN suggests they may rely on more complex nitrogen sources. Desulfobacterota, on the other hand, were positively correlated with AN and SOM, highlighting their role in environments rich in both organic matter and bioavailable nitrogen [94]. However, their negative correlation with AP suggests they are less reliant on phosphorus and may thrive better in phosphorus-limited environments. These findings highlight the diverse strategies of soil microorganisms in response to varying nutrient availabilities.

### 4.3. Effects of Land Use Patterns on Soil Nitrogen Processes and Microbial Properties

The soil nitrogen mineralization process is significantly influenced by land-use types, with distinct variations observed in the activities of key nitrogen-cycling enzymes and the abundance of functional genes. Our findings reveal that AL and WL soils exhibit reduced ammonia-oxidizing activity compared with FL. These results are consistent with previous studies that suggest agricultural soils often exhibit altered nitrogen-cycling dynamics due to intensive farming practices and disturbances, which can reduce microbial efficiency in nitrogen transformation [95]. The decrease in AMO activity in AL and WL is particularly noteworthy, as it suggests that agricultural and wetland soils may not support AOB and AOA as efficiently as forest soils, potentially due to less favorable substrate availability or altered redox conditions [96].

In terms of nitrification, FL showed the highest β-G and CBH activities, aligning with earlier studies showing that forest ecosystems tend to have higher microbial activities associated with the breakdown of organic materials and nutrient cycling [97]. However, the activities of HAO, a key enzyme in the nitrification pathway, were significantly reduced in AL, indicating that the addition of fertilizers and other anthropogenic activities in agricultural soils may disrupt nitrification processes [98]. Interestingly, in contrast, the activities of NAG were significantly increased in both AL and WL compared with FL, highlighting the greater role of microbial activity related to the decomposition of chitin and organic nitrogen in these land-use types [99]. The abundance of nitrogen-cycling functional genes further supports the observed trends in enzyme activities. For instance, the abundance of the ammonia-oxidizing functional gene AOA-*amoA* was significantly higher in AL, indicating that agricultural soils strongly support ammonia-oxidizing archaea, which are often more tolerant to fluctuations in environmental conditions and nutrient availability compared with AOB [100]. In contrast, AOB-*amoA* was significantly enriched in FL, reflecting the strong proliferation of ammonia-oxidizing bacteria in forest soils, which are generally more stable in nutrient cycles and provide a consistent source of ammonium through organic decomposition [101]. Interestingly, the abundance of the denitrification genes *nirS*, *nirK*, and *napA* was consistent across FL, AL, and WL, with no significant differences, suggesting that denitrification processes may not be as strongly influenced by land-use type as other nitrogen transformations. However, the abundance of the nitric oxide reduction gene *norB* was significantly higher in AL, indicating that agricultural soils have a more active role in nitric oxide reduction, possibly due to the increased availability of nitrate from fertilization [102]. Similarly, the nitrous oxide reduction gene *nosZ* was more abundant in AL, underscoring the potential for agricultural soils to contribute to nitrous oxide reduction despite the higher risk of greenhouse gas emissions in these systems.

The nitrogen fixation functional gene *nifH* showed significantly higher abundance in FL and AL compared with WL. This suggests that forest and agricultural soils are more conducive to nitrogen fixation, likely due to the more stable and nutrient-rich environments that support diazotrophic microorganisms [103]. The higher abundance of *nifH* in FL and AL indicates that these soils maintain stable nitrogen-fixing communities, which are critical for maintaining soil fertility and reducing the need for synthetic nitrogen fertilizers [104].

Different land-use types exert varying effects on the nitrogen-cycling process and the microbial communities responsible for these transformations. Forest soils tend to support a more stable and efficient nitrogen cycle, while agricultural and wetland soils experience more fluctuating microbial activities and nitrogen transformations, driven by human interventions and environmental conditions. Sustainable land management practices, such as reduced tillage and optimized fertilizer application, could help mitigate some of these disruptions and enhance nutrient-cycling efficiency in agricultural soils.

### 4.4. Land-Use Types Impact Microbial Network Complexity, Connectivity, Robustness, and Stability

The co-occurrence network analysis across FL, AL, and WL revealed distinct patterns in both network architecture and stability. Forest soils were characterized by a relatively sparse network—lower node and edge counts—implying localized microbial interactions and reduced complexity. Although FL appeared to have a large number of peripheral nodes with fewer hubs, it also displayed high robustness and stability, suggesting that its microbial network retains functionality even if certain taxa are lost. Such stable but less-connected networks are often observed in undisturbed or less frequently disturbed ecosystems, where consistent litter inputs and stable microhabitats favor specialized yet resilient microbial interactions [105]. By contrast, agricultural soils exhibited the highest network complexity, marked by increased node and edge counts and a greater number of critical hub taxa. This indicates a highly interactive microbial community, potentially driven by regular inputs of fertilizers, crop residues, and periodic tillage that create dynamic nutrient hotspots. Although high connectivity can support rapid resource utilization and adaptation, it also leads to decreased network stability, as disturbances (e.g., heavy tillage, pesticide application) can propagate more readily through a highly integrated network [106]. The elevated robustness in certain modules of AL soils, along with their heightened vulnerability, underscore the dual nature of intensively managed agricultural systems—capable of supporting dynamic microbial processes but prone to systemic shifts under severe or repeated disturbances [107]. Wetland soils, displaying moderate complexity and connectivity, highlight a balanced microbial co-occurrence network that falls between the extremes of FL and AL. The greater proportion of connector nodes suggests that wetland microbial communities feature strong cross-module interactions, particularly under fluctuating redox and hydrological conditions [108]. Though WL exhibits a higher overall vulnerability to large-scale perturbations, likely due to the tightly interwoven nature of its microbial interactions, its natural hydrological buffer may confer resilience against typical environmental stressors, thus maintaining functional stability under moderate disturbances [109]. Taken together, these findings confirm that land use practices have profound impacts on microbial community assembly and network properties. Forest ecosystems maintain stable though less connected networks bolstered by minimal disturbances; arable lands foster extensive interactions but exhibit reduced stability due to frequent perturbations; and wetlands form intermediate communities characterized by modular connectivity yet susceptibility to large-scale disruptions [110]. Understanding these patterns is essential for devising tailored soil management strategies that bolster microbial resilience, optimize nutrient cycling, and minimize negative environmental impacts.

## 5. Conclusions

This study highlights that distinct land-use patterns—forestland, arable land, and wetland—profoundly affect soil physicochemical properties, microbial community composition, functional gene distributions, and co-occurrence network structures. Forest soils, characterized by higher total phosphorus levels and moderate microbial diversity, support a relatively sparse yet highly stable microbial network. Arable soils show marked shifts in nutrient availability and higher overall microbial interaction densities, which enhance nitrogen transformation processes but also reduce network stability due to frequent agricultural disturbances. Wetland soils exhibit elevated organic matter and specialized anaerobic microorganisms, promoting diverse but moderately interconnected communities that are adaptive to fluctuating hydrological regimes. However, their strongly connected networks can be vulnerable under large-scale disturbances. Overall, these findings emphasize that land-use management—through factors like nutrient inputs, disturbance regimes, and hydrological control—strongly regulates microbial ecology and soil nutrient cycling. Establishing practices that balance productivity with ecological sustainability can thus optimize both soil health and the resilience of microbial communities across diverse landscapes. Further research integrating multi-omics tools and long-term monitoring is recommended to unravel the dynamic interactions among soil microbial communities under changing environmental conditions, thereby providing actionable insights for more sustainable land-use and soil-management strategies.

## Data Availability

The datasets generated during and/or analyzed during the current study are available from the corresponding author on reasonable request.

## Supplementary Materials

The following supporting information can be downloaded at: https://www.mdpi.com/article/10.3390/life15030466/s1, Table S1: Primers of target genes of quantitative PCR. References [111,112,113,114,115,116,117,118,119,120,121,122,123,124,125] are cited in the Supplementary Materials.
