# Supplementary material for: Impacts of Land Use on Soil Nitrogen-Cycling Microbial Communities: Insights from Community Structure, Functional Gene Abundance, and Network Complexity
Source: Life (Basel). 2025 Mar 14;15(3):466. doi: 10.3390/life15030466 (PMC11943759; doi:10.3390/life15030466)
Supplement: Supplementary file 1 [file life-15-00466-s001.zip › life-3463910-supplementary.pdf]

Table S1. Primers of target genes of quantitative PCR.

Targeted gene	Primer	Primer sequence(5'→3')	References
AOA-*amo*A	Arch-*amo*AF	5ATAGAGCCTCAAGTAGGAAAGTTCTA	[100]
	Arch-*amo*AR	CCAAGCGGCCATCCAGCTGTATGTCC	
AOB-*amo*A	*amo*A-1F	GGGGTTTCTACTGGTGGT	[101]
	*amo*A-2R	CCCCTCKGSAAAGCCTTCTTC	
*nir*K	*nir*K876	ATYGGCGGVCAYGGCGA	[102]
	*nir*K1040	GCCTCGATCAGRTTRTGGTT	
*nir*S	*nir*SCd3aF	AACGYSAAGGARACSGG	[103]
	*nir*SR3cd	GASTTCGGRTGSGTCTTSAYGAA	
*nos*Z	*nos*Z-F	CGCTGTTCITCGACAGYCAG	
	*nos*Z-R	ATGTGCAKIGCRTGGCAGAA	
gdh	*gdh* forward	CCACTTATTGCATTTACGTCAAAGA	[104]
	gdh reverse	CCCAGTCATCTCAGCAAGAGAA	
*nif*H	*nif*HF	AAAGGYGGWATCGGYAARTCCACCAC	[105]
	*nif*HRb	TGSGCYTTGTCYTCRCGGATBGGCAT	
*nap*A	V17m	TGGACVATGGGYTTYAAYC	[106]
	*nap*A4r	ACYTCRCGHGCVGTRCCRCA	
narG	*narG*-F	AGCTCGATGCTTAGTACCTA	[107]
	narG-R	TGCATGGTACCTGACTTGGA	
norB	*norB*-F	TGCACCTACACCGGCTACAA	[108]
	norB-R	CGTGTGTCAGATCCAGCCAT	
